# Fostering public health and academic partnerships during and beyond a public health emergency: lessons learned from COVID-19

**DOI:** 10.1093/aje/kwaf007

**Published:** 2025-01-16

**Authors:** Tomás M León, Lauren A White, Hilary Spindler, Joshua Schwab, Maya L Petersen, Jason Vargo, William Wheeler, Priya B Shete, Seema Jain, James Watt, Erica S Pan, A Marm Kilpatrick

**Affiliations:** Center for Infectious Diseases, California Department of Public Health, Richmond, CA, United States; Center for Infectious Diseases, California Department of Public Health, Richmond, CA, United States; School of Medicine, University of California, San Francisco, San Francisco, CA, United States; School of Public Health, University of California, Berkeley, Berkeley, CA, United States; School of Public Health, University of California, Berkeley, Berkeley, CA, United States; Center for Infectious Diseases, California Department of Public Health, Richmond, CA, United States; Center for Infectious Diseases, California Department of Public Health, Richmond, CA, United States; Center for Infectious Diseases, California Department of Public Health, Richmond, CA, United States; School of Medicine, University of California, San Francisco, San Francisco, CA, United States; Center for Infectious Diseases, California Department of Public Health, Richmond, CA, United States; Center for Infectious Diseases, California Department of Public Health, Richmond, CA, United States; Center for Infectious Diseases, California Department of Public Health, Richmond, CA, United States; Ecology & Evolutionary Biology Department, University of California, Santa Cruz, Santa Cruz, CA, United States

**Keywords:** infectious disease modeling, government, Covid-19 response, academic partnerships, public health

***Editor’s note:** The opinions expressed in this article are those of the authors and do not necessarily reflect the views of the* American Journal of Epidemiology.

## Introduction

The COVID-19 pandemic severely strained local, state, and federal health agencies across the United States. Rapid and sometimes unexpected rises in disease transmission led to increased morbidity and mortality and associated shortages in health care staffing, testing capacity, and personal protective equipment. Modeling and advanced analytics that provided predictive information on the timing and magnitude of surges under various scenarios were urgently needed to inform public health response. However, in order to provide such insights, there were several challenges to overcome. First, prior to the COVID-19 pandemic, minimal infectious disease modeling capacity existed at state, tribal, local, and territorial health departments. At a federal level, the Centers for Disease Control and Prevention (CDC) also had limited capacity until the formation of the Center for Forecasting and Outbreak Analytics (CFA) in late 2021; even then, its capacity for locally focused modeling outputs remained restricted. Second, early in the COVID-19 pandemic, government systems struggled to ingest, manage, and report the tremendous volume of COVID-19 data, while data access for infectious disease modelers remained a barrier. Despite significant funding efforts by CDC and other agencies in support of data modernization and forecasting, challenges persisted for many public health agencies, including the California Department of Public Health (CDPH).

The CDPH addressed the need for infectious disease modeling expertise in multiple ways. First, CDPH engaged external public and private sector stakeholders and citizen scientists early on during the COVID-19 response to iteratively develop and synthesize modeling results and build internal capacity to interact with modeling outputs. This effort resulted in the formation of a Modeling and Advanced Analytics Team that both leveraged existing CDPH staff and onboarded infectious disease modelers. The team was dedicated to synthesizing externally developed nowcasts, forecasts, and scenarios and producing internal modeling and analytical tools, with a major focus on the launch and development of the California COVID Assessment Tool (CalCAT, https://calcat.cdph.ca.gov/). The CalCAT compiles available nowcasts, forecasts, and scenario models for both internal situational awareness and to share with other public health agencies, health care systems, and the public. These modeling resources—especially those at the county scale—were extremely helpful for understanding near-future health care impacts and informing policy decisions in the face of substantial uncertainty about COVID-19 biology, epidemiology, and control measure effectiveness.

However, the CDPH Modeling and Advanced Analytics Team sometimes had insufficient staff or specific expertise to answer pressing policy questions in a short timeframe, especially when evidence in the literature was lacking. Specifically, there was a need for rapid, ad hoc modeling and expert assessment on fast-breaking topics such as the potential impact of emerging viral variants and waning immunity on transmission. Acquiring these resources within CDPH and other health jurisdictions would have required significant time and effort amidst many competing priorities during an ongoing public health emergency. In contrast, many academics were already well positioned to digest the rapidly evolving literature on variant properties and immunity and synthesize the implications for modeling transmission and hospital burden. Therefore, CDPH recognized that its response would benefit from closer academic collaborations, including with the 10-campus University of California (UC) system, a large state-based institution.

The University of California Health-CDPH COVID Modeling Consortium (https://modelingconsortium.ucsf.edu/), hereafter referred as the “Modeling Consortium”, was launched in early 2021 with the backing of top leadership of both institutions to facilitate collaboration between CDPH and UC scientists on COVID-19 through 4 major areas. First, the Modeling Consortium awarded several rapid grants and contracts to UC-based investigators, enabled by state and federal funding for the pandemic response. The CDPH leadership and staff scientists provided frequent input on project progress, resulting in deliverables that were directly relevant to CDPH for pressing policy questions, including tailored nowcasts, forecasts, and scenarios that were displayed on CalCAT, as well as more traditional academic publications.^1^^-^^4^ Second, the Modeling Consortium provided UC investigators access to more granular data than publicly available for rapid analysis of research questions specifically relevant to California’s COVID-19 situation. Although data sharing challenges persisted, this finer scale data access and ongoing alignment with CDPH and its counties may have benefited model performance. In a retrospective analysis of forecasting performance, two California-specific forecasting models run by UC partners (ie, COVID NearTerm and LEMMA) outperformed the CalCAT ensemble when forecasting COVID-19 hospital census at the county level.^5^^-^^7^ Third, through mutual training opportunities, UC graduate students and postdoctoral researchers interacted more directly with public health practice, and CDPH employees gained access to academic courses and cutting-edge research to further their professional development. Finally, the Modeling Consortium hosted virtual seminars and targeted small-group meetings 1 to 2 times each month. Each seminar combined short-format presentations on a topic of urgent concern (eg, COVID-19 transmission in schools, masking effectiveness) followed by open discussion. These fora included dozens of scientists from across the UC system and a range of CDPH staff including the State Health Officer and State Epidemiologist who indicated that these meetings provided them with critical information to make urgent policy-related decisions and recommendations to state government leadership. The targeted small-group meetings included the CDPH Modeling Team and a core group of UC scientists who discussed the most urgent scientific questions that the CDPH Modeling Team was addressing. Three key examples that demonstrated the benefit of the Modeling Consortium were the prediction of hospital burden during emergence of the Delta and Omicron variants, discussion and synthesis of evidence around nonpharmaceutical interventions, and collaborative work on understanding COVID-19 transmission in K-12 schools.^3^^,^^6^

Several challenges to this collaboration were mitigated but not eliminated during the pandemic, and many lessons were learned from this joint effort (Table 1). It was challenging for CDPH to establish data use agreements (DUAs) quickly and to subsequently make that data available to academic investigators in real time. Provisioning data and maintaining data pipelines requires health department personnel time and resources not traditionally covered by grants and other funding mechanisms. Future efforts should include support for this necessary aspect of collaboration with contingencies for changes in data sharing depending on whether a statewide public health emergency declaration order is in effect. Health departments should also be encouraged to engage in robust and ongoing data governance practices, so that data sharing questions can be quickly evaluated and approved when needed. Another key challenge was that incentives in state government and academia were not initially aligned; cooperation depended on both academics and CDPH setting aside other activities to focus on California’s COVID-19 response. Modeling Consortium academics set aside or delayed writing publications to focus on providing model forecasts that were displayed on CalCAT, updated daily, and could be adjusted in response to feedback from CDPH leadership. The mutual engagement and singular focus in the early pandemic years paid large dividends but were unsustainable at that level beyond the crisis phase of the pandemic.

**Table 1:** Modeling consortium successes, challenges, and opportunities in preparedness and response activities.

	Preparedness	Response
**Accomplished**	Stronger trust, partnerships, and networks between academics and government staff	Collaboration on emerging topics
	Increased internal CDPH capacity for advanced modeling and analytics	Regularly scheduled seminars and targeted small-group meetings for exchange of ideas and information
	UC system academic training opportunities for CDPH staff	Funding of rapid grants and contracts for UC investigators with ongoing engagement with project deliverables from CDPH staff
	Applied public health training opportunities for academic trainees	Generation of tailored nowcasting/forecasting/scenario deliverables specific to California context
		Sharing more granular data for UC investigators via secure server
**Needs improvement**	Rapid deployment of data use agreements	Faster engagement in cryptic phase
	Health department personnel and capacity for data pipeline maintenance and provisioning	Engagement with broader interdisciplinary fields
	Incentives for public health service for academics	Better mechanisms for sharing data with academic collaborators in real time with and without a public health order in effect
		Sustainability of collaborative pandemic response

The most important outcome of the Modeling Consortium was stronger collaborations—trust, partnerships, and networks—between state-based academic and government institutions that are both focused on serving the health and well-being of Californians. Funding academics to work directly with a state health department on locally important public health problems is one way to continue to increase collaborations and improve coordination across levels and sectors. However, the feasibility and sustainability of such an approach is not certain, as the COVID-19 pandemic response facilitated funding this type of collaboration in an unprecedented way. State, territorial, local, and tribal (STLT) governments are generally not in a position to provide such funding, but funding bodies should consider alternative mechanisms wherein STLTs can help shape grant priorities and deliverables rather than being more passive partners. Building internal capacity for modeling infectious diseases within CDPH was also a key feature in promoting cross-sector communication and collaboration; the CDPH Modeling Team played a key role in translating requests from leadership to academic partners and synthesizing evidence from academic deliverables for leadership, thus amplifying the ability of partners to influence policy decisions and resource prioritization. Our experiences working together have demonstrated tangible benefits for both sides: academic trainees better understood and contributed to applied public health practice, and research was catalyzed to have real-world impact in the hands of practitioners and policymakers. Additionally, government workers were informed about cutting-edge research, while learning methodologies and approaches from academic colleagues. This training fostered workforce development and employment opportunities in public health that benefited CDPH and public health more broadly. Expanding academic incentives for this sort of public service and supporting publication of research for academic and public health scientists will support ongoing partnerships.

A key overall question is: what parts of the Modeling Consortium, and academic-public health modeling partnerships in general, should be preserved and fostered for work on current and emerging public health issues as well as pandemic preparedness going forward? CDPH occupies a unique and privileged position in having access to multiple world-class universities within the state of California. Since the number of public health jurisdictions greatly exceeds the number academic centers with expertise in infectious disease modeling, national and regional coordination will be necessary for optimizing the performance and impact of such collaborations. Some of this work is just beginning through national scenario modeling and forecasting hubs, as well as dedicated efforts such as the National Outbreak Analytics & Disease Modeling Network (Insight Net). More work is needed to make sure that such collaborations are benefiting jurisdictions across a range of capacities and experience in infectious disease modeling. As others have suggested, high-frequency, real-time outbreak analyses are well-suited tasks for in-house modeling teams.^8^ Therefore, modeling pipelines and tools should be built in a generalizable way that can be further customized to local health public context for improved performance, as circumstances allow. To support this approach, many Modeling Consortium investigators participated in the national modeling hubs, and the CDPH Modeling Team engaged in dialogs with researchers about tooling that could be used by other jurisdictions.

Pandemic and public health emergency preparedness are widely shared priorities across sectors and jurisdictions. The successes of the Modeling Consortium in California during the COVID-19 pandemic highlight the potential value of synchronized academic-government partnerships in the face of new public health threats. Increased internal public health capacity for modeling and analytics, more rapid data sharing frameworks, funding mechanisms to enable rapid support for academic efforts, and incentives to facilitate close coordination between academic and government institutions will enable a better evidence-based public health response in California and throughout the United States during the next public health emergency.
